# Effect of Morning vs. Evening Turmeric Consumption on Urine Oxidative Stress Biomarkers in Obese, Middle-Aged Adults: A Feasibility Study

**DOI:** 10.3390/ijerph17114088

**Published:** 2020-06-08

**Authors:** Cynthia Blanton, Barbara Gordon

**Affiliations:** Department of Nutrition and Dietetics, Idaho State University, Pocatello, ID 83209, USA; gordbarb@isu.edu

**Keywords:** turmeric, oxidative stress, circadian, urine, inflammation

## Abstract

The circadian rhythm of biological systems is an important consideration in developing health interventions. The immune and oxidative defense systems exhibit circadian periodicity, with an anticipatory increase in activity coincident with the onset of the active period. Spice consumption is associated with enhanced oxidative defense. The objective of this study was to test the feasibility of a protocol comparing the effects of morning vs. evening consumption of turmeric on urine markers of oxidative stress in obese, middle-aged adults. Using a within-sample design, participants received each of four clock time x treatment administrations, each separated by one week: morning turmeric; evening turmeric; morning control; evening control. Participants prepared for each lab visit by consuming a low-antioxidant diet for two days and fasting for 12 h. Urine was collected in the lab at baseline and one-hour post-meal and at home for the following five hours. The results showed that the processes were successful in executing the protocol and collecting the measurements and that participants understood and adhered to the instructions. The findings also revealed that the spice treatment did not elicit the expected antioxidant effect and that the six-hour post-treatment urine collection period did not detect differences in urine endpoints across treatments. This feasibility study revealed that modifications to the spice treatment and urine sampling timeline are needed before implementing a larger study.

## 1. Introduction

The health benefits of spice consumption are increasingly identified and described as biomedical research technologies progress. Knowledge of the medicinal properties of spices documented in historic texts [1] has developed into a growing understanding of the molecular structure and actions of the compounds in spices [2]. Current studies describe the protective effects of culinary spices and herbs against oxidative damage, inflammation, cancer, infection and neurodegeneration [3,4,5,6]. These protective effects are often, but not solely, attributed to the high concentration of bioactive polyphenols in spices [7]. For example, curcumin, a polyphenolic yellow pigment in turmeric (*Curcumin longa L.*), interacts with cell molecular targets to modulate enzyme function, upregulate tumor-suppressor genes and inhibit inflammatory signaling pathways [8,9,10]. Intervention studies of curcumin consumption and supplementation demonstrate significant reductions in biomarkers of oxidative stress and inflammation in cardiac patients [11,12]. Protection against immune activation involving the chronic release of pro-inflammatory cytokines and reactive oxygen/nitrogen species is important because these molecules damage cellular membranes, DNA and proteins, resulting in diseases such as metabolic syndrome, atherosclerosis, cancer and diabetes [13,14,15]. Spices exert their anti-inflammatory, antioxidant actions by modulating the gene expression and activity of enzymes and signaling proteins in molecular pathways of immune response [5,15,16,17,18,19,20,21,22].

The beneficial properties of dietary spices resulting from their molecular interactions with genes could be increased by synchronizing spice consumption with the peak time of gene transcription. Nearly half of protein-coding genes demonstrate circadian rhythms, with biphasic peak transcription times occurring in the hours before waking and night [23]. The field of pharmacologic chronotherapy seeks to align the administration of medication with circadian rhythms to maximize therapeutic effects and reduce adverse effects [24,25]. For example, the administration of aspirin at nighttime vs. morning is significantly more effective in reducing blood pressure in hypertensive patients [26]. Similarly, a body of evidence shows that metabolic health is protected by consuming the majority of kilocalories during the day, when molecular pathways supporting nutrient assimilation and oxidation are upregulated [27,28,29,30,31]. In this context, it is reasonable to propose that the timing of spice consumption to coincide with target molecular processes could improve the magnitude of beneficial outcomes. Biomarkers of inflammation and oxidative stress are appropriate outcomes to measure, since molecular activity of these processes display circadian rhythms [32,33,34]. Singh et al. [35,36] showed clear diurnal rhythmicity in the plasma levels of malondialdehyde (MDA, a measure of lipid oxidation) and activity of the antioxidant enzymes superoxide dismutase, catalase, glutathione peroxidase and glutathione reductase in healthy men and women. Thosar et al. [37] also reported circadian rhythms in plasma MDA, with levels rising in the morning and remaining high during the day. The rise in levels of oxidative stress during the day vs. night in humans reflects high metabolic activity and the production of oxidative species during the active phase. The circadian pattern of the increased synthesis of oxidative defense enzymes beginning in the morning is thought to be a conserved mechanism by which the body is prepared to cope with oxidative stress [32].

Turmeric and its main bioactive component curcumin are among the most studied spices in relation to health. Controlled human trials and animal experiments show significant benefits of curcumin on multiple outcomes [38,39,40,41], including endothelial function [42,43], inflammation [44,45,46], oxidative stress [47] and the development of diabetes type II [48]. Low-dose (80 mg/d) curcumin for 4 weeks produced direct and indirect effects on oxidative status in healthy adults by increasing salivary radical scavenging capacity and plasma catalase activity [49]. In cellular models, curcumin treatment suppresses free radical production and increases expression of antioxidant enzyme genes [50]. Some of the antioxidant effects of curcumin involve the modulation of cellular molecular targets, such as toll-like receptors and nuclear factor kB proteins of macrophages, which control the release of inflammatory factors [51,52]. Weisberg et al. [53] reported significant reductions in hepatic nuclear factor kB activity and the macrophage production of inflammatory molecules in mice fed 3% curcumin for five weeks. Turmeric and non-curcuminoid components of turmeric also exert bioactivity. Kim et al. [54] demonstrated a superior effect of turmeric (containing 7.9% curcumin) over an equivalent concentration of curcumin in blocking the proliferation of multiple cancer cell lines. In a study of diabetic rats, 0.5% turmeric in the diet exerted a similar, and in some measures, greater, protective effect against oxidative stress and cataract progression than did the corresponding concentration of curcumin, 0.01% [55]. Burhmann et al. [56] showed the significant inhibition of proliferation of cancer cells treated with Calebin A, a non-curcumin compound in turmeric. This inhibition was the result of Calebin A’s suppression of nuclear factor kB- and STAT3-induced tumor growth. These examples of the potent functional properties of turmeric, curcumin and other spices [57] support the focused investigation of their application in integrative functional nutrition and medicine.

An important, yet underappreciated, aspect of functional nutrition therapy is the optimal timing of consumption/administration of the food/compound [58]. The metabolic benefits of consuming food during the day vs. night are reported [59,60,61,62,63], but no known studies have addressed the impact of timed spice/herb intake on health outcomes. This feasibility study evaluated the practicality of a protocol testing for differential antioxidant effects of consuming a spice-rich food in the morning vs. in the evening. The feasibility components evaluated included participant recruitment and adherence to protocol, the flow of laboratory visit processes, the appropriateness of treatments and outcome measure methods, and the ability to collaborate with other laboratories performing sample analyses and adequacy of resources. Turmeric was selected as the test spice due to its reported efficacy in reducing markers of inflammation and oxidative stress [44,64,65,66,67,68]. Inflammation and oxidative stress are closely related in the pathogenesis of chronic disease [69].

## 2. Materials and Methods

### 2.1. Study Design

This study was conducted in accordance with the Declaration of Helsinki and the protocol was approved by the Institutional Review, Board Human Subjects Committee at Idaho State University, protocol number IRB-FY2019-248. All participants provided their informed consent before they started the study. The protocol consisted of four laboratory visits, each separated by one week. Each participant received all treatments and all orders of treatments. During each visit, participants provided a baseline urine sample and then consumed a test meal. Urine was collected in the laboratory at one hour post-meal and at home for the following five hours. Urine markers of oxidative stress were quantified and compared across treatments. The schedule for one study visit is shown in Table 1.

### 2.2. Participants

Obese (body mass index (BMI) 30–40 kg/m^2^) men and women aged 40–65 years were recruited from the local population using university electronic bulletin boards and paper flyers. The selection of middle-aged, obese individuals was based on evidence that this population has elevated levels of blood and urine inflammatory and oxidative stress markers [68,70,71,72]. Thus, the probability of detecting significant treatment-associated reductions in oxidative stress markers is presumably higher in an obese, middle-aged relative to normal-weight, young adult participant sample. Exclusion criteria were the inability to consume egg- or turmeric-containing food; the self-reported presence of acute infection or chronic inflammatory disease; smoker; heavy aerobic exerciser; alcohol intake > 1 drink/day; pregnancy or lactation; the use of non-steroidal anti-inflammatory drugs or dietary supplements containing > 2x RDA for vitamins/minerals or botanicals/herbs/spices.

Potential participants who contacted the research study screening staff member completed a scripted telephone screening questionnaire to assess eligibility. Those who passed the telephone screening then completed an in-person lab visit with the principle investigator (PI), that included a detailed description of the study, completing the informed consent process, instruction on following a low-antioxidant diet and keeping food records, and measurements of height and weight. Height was measured to the nearest 0.1 cm using a stadiometer and weight was measured to the nearest 0.1 kg using a beam scale. Body mass index was calculated as (weight in kg)/(height in meters)^2^.

Participants were instructed to follow a low-antioxidant diet for two days prior to each lab visit. This was included in the protocol, in order to reduce the impact of the recent intake of high-antioxidant foods and beverages on the outcome measures of oxidative stress [73]. The list of foods to avoid on the low-antioxidant diet are found in Appendix A and this was based on the literature describing antioxidant content of foods [74,75,76]. The PI examined the food records at the beginning of each lab visit to confirm participant adherence to the diet during the prior two days.

### 2.3. Dietary Assessment

At the first lab visit, during the first hour post-treatment, participants answered a diet history questionnaire to provide data on usual dietary intake. The PI administered to participants the National Cancer Institute’s online Diet History Questionnaire version III (DHQ III, National Institutes of Health, Applied Research Program, National Cancer Institute, Bethesda, MD, USA, 2018) [77], examining the past year with serving sizes. Output data from the DHQ III included Healthy Eating Index-2015 (HEI-2015) total and individual component scores and average daily nutrient intakes. The HEI-2015 assesses diet quality according to adherence to the USA Dietary Guidelines, with scores ranging from 0 (no adherence) to 100 (optimal adherence).

Participant food records were entered into the National Cancer Institute’s online Automated Self-Administered 24-Hour Dietary Assessment Tool (ASA24^®^, version 2018) [78], developed by the National Cancer Institute, Bethesda, MD, USA. Food records were analyzed by ASA24 using the Food and Nutrient Database for Dietary Studies (FNDDS) 2011–2012 and 2013–2014 [79]. Output data from the ASA24 included daily nutrient intakes.

### 2.4. Treatments

Two test foods were provided at two clock times: spiced morning; spiced evening; no spice morning; no spice evening. The order of treatment and time were counterbalanced across participants and each participant was randomly assigned to an order/time combination. Scrambled pasteurized egg white was used as the spice vehicle, in order to minimize the number of nutrient components present in food/beverages that influence inflammatory/oxidative stress status [80,81]. Egg white generally has a neutral effect on inflammatory/oxidative stress biomarker levels [82] and it is acceptable for consumption at morning and evening meals in the United States. Egg yolk was excluded due to its high content of carotenoids, which have antioxidant properties that would confound the study’s results. The spiced food contained 180 mL egg white (Lucerne Foods Inc., Boise, 83706, ID, USA) and 5 g ground turmeric (McCormick & Company Inc, Hunt Valley, 21031, MD, USA) and was cooked in a microwave oven for approximately 3 min immediately before serving. Ground turmeric, prepared from dried *Curcuma longa* rhizomes, was chosen for this study in order to reflect a culinary source of curcumin that is readily available to consumers in the United States. Ground turmeric contains 3–15% curcuminoids [83]. The no-spice food contained 180 mL egg white and no turmeric. The food was served with 500 mL water to 12-h fasted participants.

Morning (7:00 A.M.) and evening (6:00 P.M.) were chosen as the test food administration clock times, based on findings that circadian rhythms in many physiological systems such as gene expression occur according to the day-night cycle [23]. A stronger antioxidant effect of the morning compared to evening spiced food was hypothesized considering the gradual increase in plasma malondialdehyde concentrations in the morning [35,37].

### 2.5. Urine Collection and Biomarker Analysis

A baseline urine sample was collected at each laboratory visit prior to test meal consumption. For the 6-h period following test meal consumption, participants were instructed to drink water only, and to eat no food. A 1-h post-meal urine sample was collected in the laboratory before participants left for work/home, where they collected urine in batches at post-meal hours 1–2, 2–4 and 4–6. The urine collection schedule was based on published data demonstrating that biomarker levels of oxidative stress show peak change due to spice intake within 5 h of consumption [65].

Urine samples collected outside the laboratory were stored in a refrigerator or temporarily placed in a cooler before transfer to a refrigerator. Participants delivered home-collected samples in a cooler to the investigator within 12 h of the 6-h sample collection for storage at −80 °C prior to analysis.

Urine was analyzed for markers of oxidative stress, malondialdehyde (MDA) and 8-isoprostanes [84,85,86] using commercial kits. MDA was quantified using a colorimetric assay purchased from Cayman (TBARS assay kit, item number 700870; Cayman Chemical, Ann Arbor, 48108, MI, USA). Urine 8-isoprostane was measured using an enzyme-linked immunoassay kit (8-isoprostane ELISA kit, item number 516351; Cayman). Concentrations of MDA and 8-isoprostane were normalized to urine creatinine concentration. Creatinine was quantified using a colorimetric assay kit (Creatinine urinary colorimetric assay kit, item number 50070, Cayman). The time points used for analysis were hours 0, 0–2, 2–4, and 4–6.

### 2.6. Statistical Analysis

General linear models (GLM) were used to predict urine biomarker levels over time point by treatment and clock time. Biomarker values were not normally distributed and thus they were log-transformed prior to analysis. Descriptive statistics are reported as mean with standard deviation and median with interquartile range. The fixed effects of treatment (spice vs. no spice), clock time (A.M. vs. P.M.), time point (hour 0, 0–2, 2–4, and 4–6) and their interactions on biomarker level and biomarker level as a percent of baseline were tested. Participant was included as a random effect in the models. When significant main effects were seen, post-hoc tests were conducted using Tukey’s multiple comparisons test for time point and student’s t-test for treatment and clock time. Dietary intakes of antioxidant micronutrients, including vitamins A, C and E and the mineral selenium were calculated from the diet history questionnaire, which assessed baseline diet, and food records, which assessed intake during the low-antioxidant diet periods. Antioxidant intakes were log-transformed due to non-normal distribution and compared between methods using GLM, with participant included as a random variable.

### 2.7. Assessment of Feasibility of Study Protocol

Feasibility of the study protocol was evaluated within these categories: (1) participant recruitment, retention and adherence to protocol; (2) flow of laboratory visit processes; (3) appropriateness of dietary assessment methodologies; (4) appropriateness of treatments and urine collection methods for detecting, at minimum, a trend showing a treatment effect; (5) ability to collaborate with another laboratory for sample analysis; and (6) adequacy of resources. In addition, sample sizes for a main study were calculated using the biomarker means and standard deviations obtained in this feasibility study.

## 3. Results

Results of the study outcomes are presented first followed by an evaluation of the feasibility of the study components.

### 3.1. Participant Characteristics

Ten people responded to the study advertisement within two months of initiation and six were excluded due to body mass index below range and/or scheduling conflicts. Four participants who met the study criteria were enrolled and completed the full study. Participant (*n* = 4, 3 male) body mass index and age (median and range) were 33.3 kg/m^2^ (31.4–34.0) and 60.5 y (53–63). The usual dietary intake of the participants is shown in Table 2. Participants’ HEI-2015 total scores (66.7 ± 9.6 (mean ± standard deviation) were higher than the U.S. average for the 18–64 year-old age group (HEI = 58) and the 65 + year-old group (HEI = 64) [87].

Participant adherence to the low-antioxidant diet (the two days preceding each laboratory visit) was confirmed by the examination of food records, which showed avoidance of high-antioxidant foods and beverages and selection of the recommended low-antioxidant items. Intakes of select antioxidant micronutrients during the low-antioxidant diet periods are shown in Table 3. Compared to baseline, the participants consumed significantly smaller amounts of vitamins A, C and E during the low-antioxidant periods. Intakes of vitamins C and E were below the recommended dietary allowance values during the low-antioxidant periods, but not during the baseline diet.

### 3.2. Biomarker Response to Treatment

**Figure 1:**
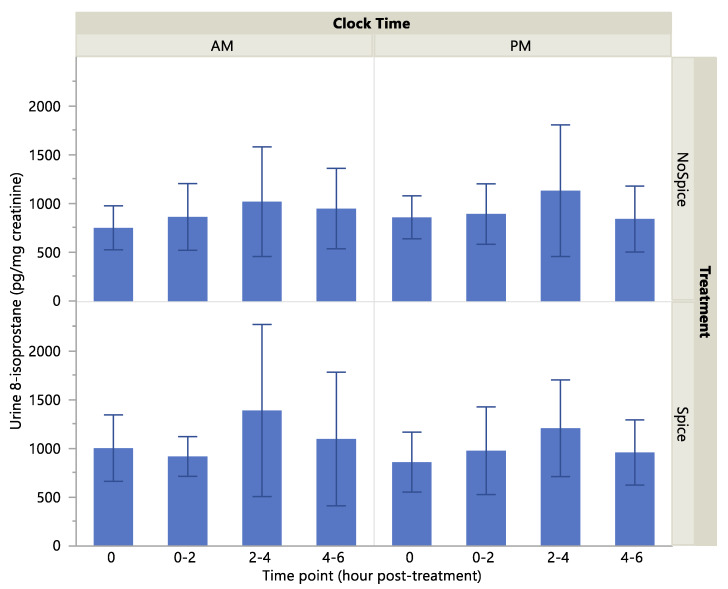
Urine 8-isoprostane concentrations by treatment, clock time and time point. Values are means with standard deviation. Effect of treatment, *p* = 0.02; clock time, *p* > 0.05; time point, *p* = 0.03.

**Figure 2:**
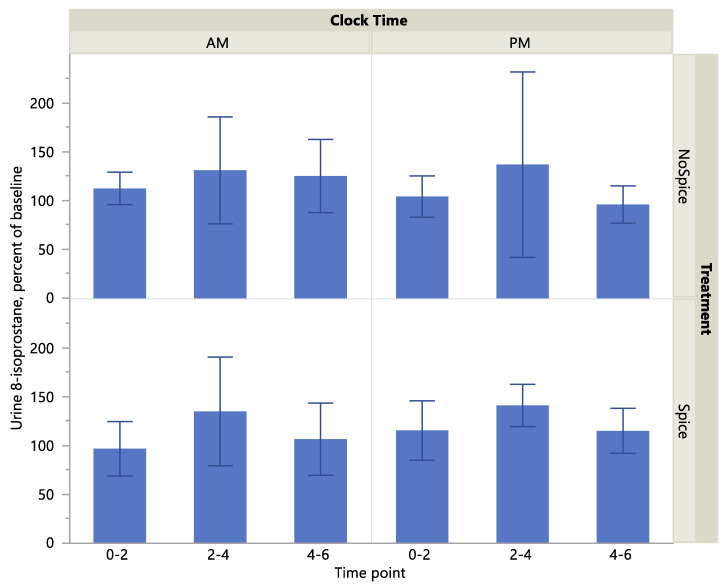
Urine 8-isoprostane as percent of baseline by treatment, clock time and time point. Values are means with standard deviation. Values tended to be higher at the 2–4-h compared to 0–2- and 4–6-h time points (*p* = 0.05), but showed no significant effect of treatment or clock time (*p* > 0.05).

Urine 8-isoprostane concentration was significantly higher following consumption of the spice vs. no spice meal (*p* = 0.02) and at the 2–4-h compared to the hour 0 (before treatment) time point (*p* = 0.03), Table 4. Clock time (A.M. or P.M.) did not have a significant effect on 8-isoprostane level (*p* > 0.05). Urine 8-isoprostane level as a percent of baseline tended to be higher at the 2–4-h compared to 0–2- and 4–6-h time points (*p* = 0.05), but showed no significant effect of treatment or clock time (*p* > 0.05). Histograms of absolute and percent-of-baseline measures of 8-isoprostane are presented in Figure 1 and Figure 2. Cumulative (post-treatment hours 0–6) urine concentrations of 8-isoprostane did not differ significantly according to clock time or treatment or their interaction (*p* > 0.05; data not shown).

**Figure 3:**
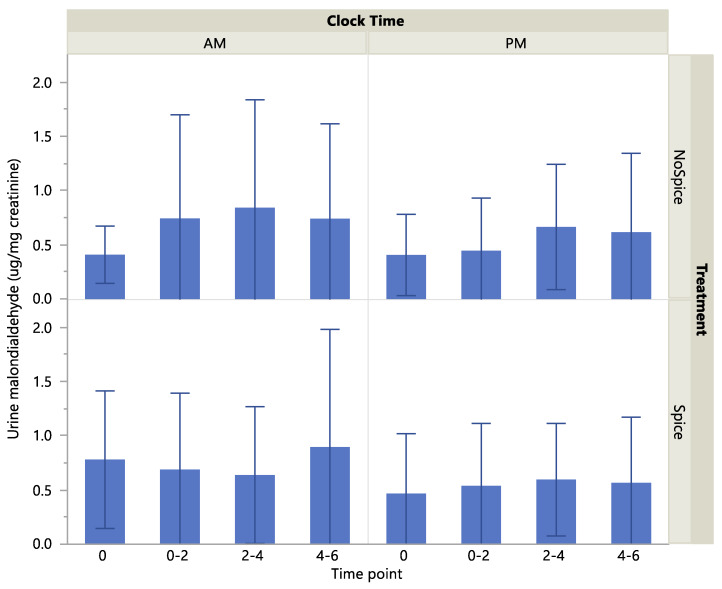
Urine malondialdehyde concentrations by treatment, clock time and time point. Values are means with standard deviation. Effect of treatment, *p* > 0.05; clock time, *p* < 0.01; time point, *p* < 0.05.

**Figure 4:**
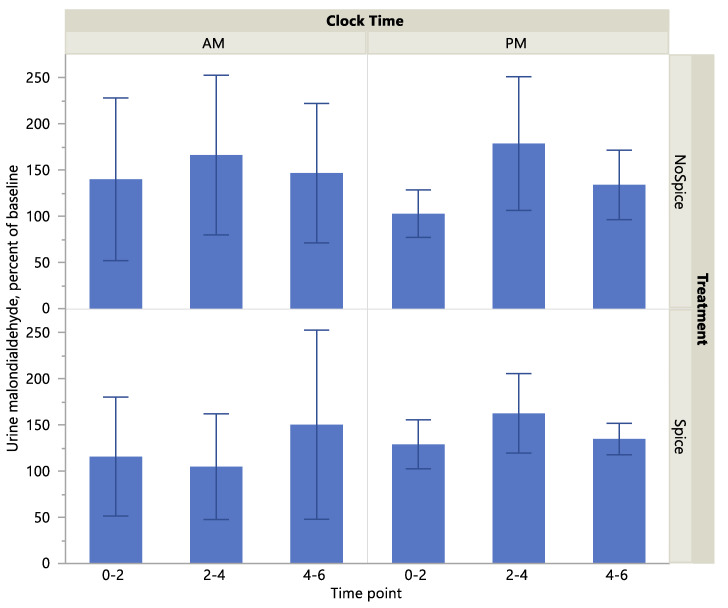
Urine malondialdehyde as percentage of baseline by treatment, clock time and time point. Values are means with standard deviation. **There was no significant effect of treatment, clock time or time point.**

**Figure 5:**
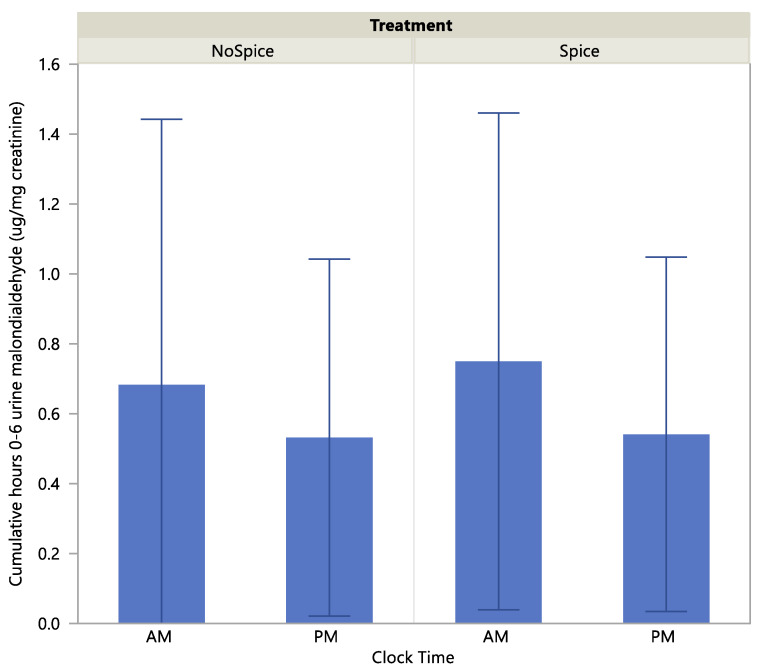
Cumulative hours 0–6 urine malondialdehyde by treatment and clock time. Effect of clock time, *p* < 0.05; treatment, *p* > 0.05; clock time x treatment interaction, *p* > 0.05.

Urine malondialdehyde level was significantly higher at the A.M. vs P.M. clock time (*p* < 0.01) and at the 2–4-h compared to the hour 0 time point (*p* < 0.05) Table 5. Treatment did not have a significant effect on malondialdehyde level (*p* > 0.05). Malondialdehyde as a percent of baseline showed no significant effect of treatment, clock time or time point. There were no significant interactions across treatment, clock time and time point on either biomarker level. Scatterplots of absolute and percent-of-baseline measures of malondialdehyde are presented in Figure 3 and Figure 4. Cumulative urine concentrations of malondialdehyde were significantly higher in the A.M. vs P.M. (*p* < 0.05; Figure 5), but did not differ significantly according to treatment or a clock time x treatment interaction (*p* > 0.05).

### 3.3. Assessment of Feasibility of Study Protocol

Participant recruitment methods utilizing electronic bulletin boards and paper flyers were effective in securing the small number of people needed for this feasibility project. The participants adhered to protocol activities and completed the study. The participants followed instructions correctly for the schedule of laboratory visits, dietary restrictions, food intake recording, urine collection, and urine collection recording. The treatments were well tolerated and participants reported no negative aspects of the protocol, except needing to avoid coffee before morning study visits and during the low-antioxidant diet period. Participants stated that monetary compensation provided motivation to enroll and complete the study.

The flow of processes composing the laboratory visits was appropriate for obtaining the data as planned. The time allotted for each procedure and laboratory visit was adequate, with the exception of the first visit, when the Diet History Questionnaire was administered. The questionnaire required approximately 60 min to administer, which would have been better accommodated by involving several research staff members or extending the scheduled duration of the first visit, especially with concurrent appointments. Urine collection was completed as expected, with participants using the labeled containers appropriately and depositing home-collected samples at the correct time and place.

Dietary assessment using online tools was successful in this feasibility study. By administering the Diet History Questionnaire (DHQ III) in person to participants, the accuracy of responses was optimized. In the investigators’ experience, in-person administration of a dietary assessment tool is superior to self-administration for collecting valid data. The kilocalorie and nutrient results obtained in this study were within expected ranges and thus support their validity. Further, the DHQ III, as well as the ASA24 program used to analyze the food records, automatically generated full nutrient intake data. This was important for reducing time and cost expenditures for coding and analyzing dietary information.

The absence of a treatment effect on the urine markers of oxidative stress indicates that modifications to the treatment and/or outcome measure methodology are needed before proceeding with a main study. The consumption of 5 g of turmeric, which was confirmed by direct observation, elicited no consistent suppressive effect on two measures of oxidative stress, 8-isoprostane and malondialdehyde. The fact that the assays produced biomarker measurements within ranges seen in the literature and detected time-point and clock-time variations in values suggests that the tests were performed properly.

Collaborating with a core laboratory on the same university campus was effective for analyzing the urine samples for oxidative stress markers. Cost savings were realized by having experienced scientists perform the assays in a fully equipped laboratory at a reasonable rate. The feasibility study demonstrated that the time and laboratory resources needed for assay optimization, such as determining dilution ratios, must be incorporated into the budget and timeline.

Overall, resources were appropriately budgeted and planned, as shown by completion of the pilot study within budget and schedule. Minor adjustments were needed to accommodate the time needed for study visit 1 and the time and supplies required for assay optimization. In a larger study it would be important to determine the number of men and women enrolled before purchasing urine collection supplies, since urine collection pans, for example, are useful only for females.

Sample sizes for a cross-over design main study were calculated using the biomarker data obtained in this feasibility study. Results showed that 18 and 23 participants would be needed to detect a statistically significant effect of treatment on urine 8-isoprostane level with 80% and 90% power, respectively. For MDA, 28–37 participants would be needed to detect an effect of treatment with 80% and 90% power, respectively.

## 4. Discussion

This feasibility study evaluated the outworking of a protocol testing the effect of clock time (morning vs. evening) on oxidative stress biomarker levels following the consumption of a turmeric-rich meal. Findings were that participants followed protocol and completed the study, processes were appropriately planned and executed for collecting data, collaboration with another laboratory was effective in obtaining biomarker measurements and resources were appropriately budgeted. However, a major concern was raised in the lack of an effect of the spice treatment on biomarker outcome measures. Although this was a pilot study in which statistically significant effects of treatment were not expected due to insufficient power, a trend showing an effect was anticipated. Testing the hypothesis that clock time affects biomarker response to turmeric is contingent on showing a general suppressive effect of turmeric on oxidative stress markers. Thus, a delivery method of turmeric that produces an antioxidant effect must be identified before advancing to a main study. The rationale for the hypothesis was based on evidence showing circadian rhythmicity in blood and urine levels of oxidative stress markers [88] and findings that turmeric reduces oxidative stress markers [46,89].

A modification to the treatment that may elicit an antioxidant effect is to combine turmeric with a known enhancer of bioavailability, piperine in black pepper (*Piper spp*.) Piperine inhibits small intestinal glucuronidation of curcuminoids and other polyphenols, resulting in their increased bioavailability [90,91,92]. Changing the vehicle for the turmeric + piperine treatment from egg white to a high-fat, high-carbohydrate meal may further increase the likelihood of detecting a spice effect. A high-fat, high-carbohydrate meal is shown to provoke oxidative stress and an inflammatory response in humans [93,94,95,96]. In the context of such an oxidative stress challenge, previous investigators have demonstrated significant antioxidant effects of spices [96] and fruit polyphenols [93,94].

Another protocol modification that would improve the probability of detecting an antioxidant effect of the spice treatment is to expand the urine collection period to 24 h. The six-hour post-treatment urine collection period may have been insufficient to identify antioxidant effects over an appropriate time scale. By analyzing 24-h post-treatment urine samples, cumulative measurements of the oxidative stress marker may reveal differences between treatment groups.

Beyond identifying concerns regarding the appropriateness of the treatment and urine collection methods, this feasibility study validated multiple components of the protocol. First, a sufficient number of participants were recruited in a timely manner using electronic bulletin boards and posted flyers. To enroll a larger number of participants within a reasonable period of time, however, the targeted recruitment of eligible people from a medical patient database would likely be necessary. Second, the participants adhered to instructions and completed the full study. The cross-over design allowed for the comparison of treatment effects within individuals, thereby reducing the impact of confounding variables. Adherence to the low-antioxidant diet was confirmed by the analysis of participants’ food records. Notably, the mean intakes of 10.7 (7.8) mg vitamin C and 8.8 (8.6) mg vitamin E during the low-antioxidant diet in the current study were similar to or lower than those of participants consuming a low-total antioxidant capacity diet in a study conducted by Valtuena et al. [97]: 91.7 (72.1) mg vitamin C and 7.5 (1.6) mg vitamin E.

Third, the protocol process flowed well, as demonstrated by the successful execution of the planned actions and the collection of target data. Instruction booklets and study activity calendars appeared to be valuable for guiding participants in their responsibilities. Clear labeling of urine collection containers with verbal instructions for use were also important for participant adherence. Reminder phone calls and emails resulted in 100% on-time attendance at laboratory visits and sample deposit meetings.

Fourth, collaboration with the university’s Molecular Research Core Facility was effective in completing the urine analyses. The laboratory is equipped and its staff are experienced to perform the assays at a reasonable cost for investigators. One limitation noted was the lack of planning for assay optimization, in which appropriate dilutions are determined and procedures are tested. A main study would need to allocate sufficient time and supplies for this important protocol component.

Fifth, resources were generally appropriately planned and utilized. The feasibility study was completed on schedule and the budget was met.

Lastly, sample size calculations for a main study using a cross-over design indicated that ~40 participants would be needed to detect statistically significant differences in both biomarker levels across treatments with 90% power.

## 5. Conclusions

In conclusion, this feasibility study identified a lack of an observable effect of turmeric on urine oxidative stress markers, which must be resolved prior to moving forward with a main study. Testing the hypothesis that turmeric consumption can be timed to optimize its oxidative defense activity requires the demonstration of an antioxidant effect of turmeric. Modifications to the spice delivery method and urine collection schedule are suggested solutions. Other components of the feasibility study protocol related to the processes, participants and collaborations were successful in completing the study and collecting the intended data.

## Figures and Tables

**Table 1:** Study schedule for one visit. Participants completed four visits, each separated by one week.

Activity	2 Days Prior to Lab Visit	12 Hours Prior to Lab Visit	Lab Visit	5 Hours Post Lab Visit
Low-antioxidant diet	x			
Food records	x			
Water-only fast		x		x
Urine sample			x (pre-treatment and 1-h post treatment)	x (batched for hours 1–2, 2–4 & 4–6 post treatment)
Treatment administered			x	
Diet history questionnaire administration			x (visit 1 only)	

**Table 2:** Usual dietary intake data of participants (*n* = 4).

HEI-2015 Output	Mean (SD)	Median (IQR)
Total HEI-2015 score (maximum = 100)	66.7 (9.6)	68.3 (18.2)
Component Scores (maximum)		
Total Vegetables (5)	4.7 (0.6)	5 (0.9)
Greens and Beans (5)	4.8 (0.5)	5 (0.7)
Total Fruits (5)	3.1 (1.4)	2.7 (2.7)
Whole Fruits (5)	3.2 (2.1)	3.6 (3.9)
Whole Grains (10)	3.5 (1.7)	4.2 (2.8)
Dairy (10)	7.1 (3.4)	7.5 (6.2)
Total Protein Foods(5)	5 (0)	5 (0)
Seafood and Plant Proteins (5)	4.9 (0.2)	5 (0.2)
Fatty Acids (10)	6.1 (3.0)	7.3 (5.1)
Sodium (10)	2.0 (2.1)	2.0 (3.7)
Refined Grains (10)	9.7 (0.4)	9.7 (0.7)
Saturated Fats (10)	3.9 (3.3)	3.8 (6.4)
Added Sugars (10)	8.7 (2.2)	9.6 (3.6)
Energy (kilocalories)	2405.2 (491.9)	2337.3 (935.3)
Energy from fat (% kilocalories)	42.4 (10.9)	43.3 (20.3)
Energy from carbohydrate (% kilocalories)	40.0 (10.7)	41.3 (20.3)
Energy from protein (% kilocalories)	19.4 (5.3)	20.2 (10.1)

Data were collected and Healthy Eating Index 2015 scores were calculated using the National Cancer Institute’s Diet History Questionnaire III assessing the past year. SD = standard deviation; IQR = Interquartile Range.

**Table 3:** Antioxidant nutrient intakes of participants during low-antioxidant diet and baseline.

	Low-Antioxidant Diet Days		Baseline Diet		*p* Value	RDA *, Age 51–70 y
	Mean (SD)	Median (IQR)	Mean (SD)	Median (IQR)		
Total vitamin A activity (RAE), mcg	1057.1 (586.3)	937.1 (1016.0)	1863.6 (560.5)	1966.0 (1056.2)	0.03	900 (males), 700 (females)
Vitamin C, mg	10.7 (7.8)	10.5 (13.6)	122.6 (15.2)	119.2 (27.8)	<0.0001	90 (males), 75 (females)
Vitamin E (alpha-tocopherol), mg	8.8 (8.6)	5.8 (6.2)	20.6 (10.5)	19.6 (19.8)	<0.01	15 (males & females)
Selenium, mcg	160.8 (74.4)	143.5 (79.5)	142.0 (31.3)	140.2 (60.5)	0.75	55 (males & females)

Low-antioxidant diet data were collected from food records kept by participants two days prior to each laboratory visit. Total number of food records per participant = 8. Baseline diet data were collected from a diet history questionnaire assessing the previous year. SD = standard deviation; IQR = Interquartile Range; RAE = Retinol Activity Equivalent; * RDA = Recommended Dietary Allowances, US Dietary Reference Intakes. Nutrient comparisons are within rows and between food records and baseline diet.

**Table 4:** Urine 8-isoprostane concentrations by treatment, clock time and time point.

**Time Point**	**Spice, A.M.**		**Spice, P.M.**	
	**Mean (SD)** **pg/mg Cr**	**Median (IQR)** **pg/mg Cr**	**Mean (SD)** **pg/mg Cr**	**Median (IQR)** **pg/mg Cr**
0 h (baseline)	1004.0 (400.0)	968.6 (653.3)	859.8 (307.9)	903.6 (574.8)
0–2 h	917.7 (204.5)	897.5 (370.9)	976.2 (450.1)	811.5 (781.5)
2–4 h	1390.7 (883.3)	1119.1 (1596.8)	1207.6 (495.7)	1125.5 (939.4)
4–6 h	1096.5 (685.9)	800.3 (1105.0)	959.5 (333.6)	879.2 (607.2)
	**No Spice, A.M.**		**No Spice, P.M.**	
0 h (baseline)	752.2 (225.3)	800.5 (411.2)	859.7 (220.1)	838.2 (423.3)
0–2 h	863.4 (342.7)	875.0 (658.1)	893.4 (310.7)	764.4 (509.7)
2–4 h	1020.4 (563.2)	1053.1 (1056.7)	1132.8 (675.7)	971.0 (1222.0)
4–6 h	949.3 (413.2)	968.3 (786.7)	842.6 (337.9)	785.5 (623.8)

General linear models analysis, effect of treatment (spice vs. no spice), *p* = 0.02; clock time (A.M. vs. P.M.), *p* > 0.05; time point (hours 0, 0–2, 2–4, 4–6), *p* = 0.03. SD = standard deviation; IQR = Interquartile Range.

**Table 5:** Urine malondialdehyde concentrations by treatment and time point.

**Time Point**	**Spice, A.M.**		**Spice, P.M.**	
	**Mean (SD)** **µg/mg Cr**	**Median (IQR)** **µg/mg Cr**	**Mean (SD)** **µg/mg Cr**	**Median (IQR)** **µg/mg Cr**
0 h (baseline)	0.78 (0.63)	0.68 (1.16)	0.47 (0.55)	0.21 (0.87)
0–2 h	0.69 (0.70)	0.36 (1.09)	0.54 (0.58)	0.26 (0.89)
2–4 h	0.64 (0.63)	0.35 (0.99)	0.59 (0.52)	0.36 (0.81)
4–6 h	0.90 (1.09)	0.45 (1.81)	0.57 (0.60)	0.29 (0.95)
	**No Spice, A.M.**		**No Spice, P.M.**	
0 h (baseline)	0.41 (0.26)	0.28 (0.40)	0.41 (0.38)	0.25 (0.62)
0–2 h	0.74 (0.96)	0.28 (1.47)	0.44 (0.49)	0.20 (0.73)
2–4 h	0.84 (1.00)	0.38 (1.57)	0.66 (0.58)	0.40 (0.92)
4–6 h	0.74 (0.88)	0.34 (1.38)	0.62 (0.73)	0.26 (1.11)

General linear models analysis, effect of treatment (spice vs. no spice), *p* > 0.05; clock time (A.M. vs. P.M.), *p* < 0.01; time point (hours 0, 0–2, 2–4, 4–6), *p* < 0.05.

## Appendix A. Instructions Provided to Participants for Following a Low-Antioxidant Diet

**Foods and beverages to avoid on the low-antioxidant diet**
**Beverages**
Tea—all types, including green, black, oolong
Alcoholic beverages including wine and beer
Coffee
Hot cocoa and chocolate milk
Fruit juices
Vegetable juices
Soy milk
**Spices—all**
Examples:
Turmeric, Curcumin
Black Pepper
Cinnamon
Oregano
**Fruits**
Apples
Apricots
Avocado
Bananas
Berries (Blackberries, Blueberries, Cranberries, Raspberries, Strawberries)
Cherries
Dates
Figs
Kiwi
Red, purple, black grapes
Prunes
Raisins
Citrus fruits (grapefruit, orange)
Mango
Peaches (raw)
Pears (raw)
Plums
**Vegetables**
Artichokes
Asparagus
Beans (small red, pinto, kidney, black)
Beets
Bell peppers (red, green, yellow, orange)
Broccoli
Cabbage
Eggplant (raw)
Green leafy vegetables (romaine, spinach, kale, collard greens)
Onions
Russet or red potato
Sweet potatoes
Tomatoes-all fresh and processed products (tomato sauce or paste, tomato juice,
spaghetti sauce, pizza sauce, salsa etc.)
**Nuts/seeds**
Nuts (such as peanuts, pecans, walnuts, hazelnuts, soynuts)
Peanut butter or other nut butters
Seeds (such as sunflower, pumpkin)
Wheat germ
**Other**
Whole-grain products
Chocolate
All soy products (soy flour, soy nuts, soy beans, soy drinks)
**Examples of allowed foods and meals**
Pancakes or waffles (not whole grain) with maple syrup
Toast, bagels, muffins (not whole grain) with margarine
Milk
Meat or cheese sandwich (not whole-grain bread)
Salad with iceberg lettuce and cucumber
Canned peaches or pears
Milk
Meat (without tomato sauce)
Pasta or rice (not whole grain)
Canned corn
White roll with butter/margarine
Water
Plain yogurt
Pretzels, chips
Celery and ranch dressing
Water
